# Machine Learning Accurately Predicts Muscle Invasion of Bladder Cancer Based on Three miRNAs

**DOI:** 10.1111/jcmm.70361

**Published:** 2025-02-10

**Authors:** Lea Eckhart, Sabrina Rau, Markus Eckstein, Phillip R. Stahl, Hiresh Ayoubian, Julia Heinzelbecker, Farzaneh Zohari, Arndt Hartmann, Michael Stöckle, Hans‐Peter Lenhof, Kerstin Junker

**Affiliations:** ^1^ Center for Bioinformatics, Saarland Informatics Campus Saarland University Saarbrücken Germany; ^2^ Department of Urology and Pediatric Urology Saarland University Medical Center and Saarland University Homburg Germany; ^3^ Institute of Pathology University Hospital Erlangen Erlangen Germany; ^4^ Institute of Pathology Saarland University Medical Center and Saarland University Homburg Germany; ^5^ Department of Medicine MSB Medical School Berlin Germany; ^6^ Department of Urology and Pediatric Urology Saarland University Homburg Germany

**Keywords:** machine learning algorithms, microRNA, molecular subtypes, muscle‐invasive bladder cancer, pT1 high‐grade tumours

## Abstract

The aim of this study was to validate the diagnostic potential of four previously identified miRNAs in two independent cohorts and to develop accurate classification models to predict invasiveness of bladder cancer. Furthermore, molecular subtypes were investigated. The miRNAs were isolated from pTa low‐grade (lg) (*n* = 113), pT1 high‐grade (hg) (*n* = 133) and muscle‐invasive bladder cancer (MIBC) (*n* = 136) tumour tissue samples (FFPE) after either transurethral resection of a bladder tumour (TURB) or cystectomy (CYS). In both cohorts, the expression of miR‐138‐5p and miR‐200a‐3p was significantly lower, and the expression of miR‐146b‐5p and miR‐155‐5p was significantly higher in MIBC compared to pTa lg. A k‐nearest neighbours (KNN) classifier trained to distinguish pTa lg from MIBC based on three miRNAs achieved an accuracy of 0.94. The accuracy remained at 0.91 when the classifier was applied exclusively to the TURB samples. To guarantee reliable predictions, a *conformal prediction* approach was applied to the KNN model, which eliminated all misclassifications on the test cohort. pT1 hg samples were classified as MIBC in 32% of cases using the KNN model. miR‐146b‐5p, miR‐155‐5p and miR‐200a‐3p expressions are significantly associated with particular molecular subtypes. In conclusion, we confirmed that the four miRNAs significantly distinguish MIBC from NMIBC. A classification model based on three miRNAs was able to accurately classify the phenotype of invasive tumors. This could potentially support the histopathological diagnosis in bladder cancer and therefore, the clinical decision between performing a radical cystectomy and pursuing bladder‐conserving strategies, especially in pT1 hg tumors.

**Abbreviations**
CP: conformal prediction
CYS: cystectomy
KNN: k‐nearest neighbours
MIBC: muscle‐invasive bladder cancer
NMIBC: non‐muscle‐invasive bladder cancer
pT1 hg: pT1 high grade
pTa lg: pTa low grade
TURB: transurethral resection of bladder tumour
VNN: vanilla neural network

## 1 Introduction

In contrast to non‐muscle‐invasive bladder cancer (NMIBC), muscle‐invasive bladder cancer (MIBC) is an aggressive tumour disease with a higher metastatic risk and worse outcomes. Therefore, radical cystectomy is the standard of care in MIBC, whereas local tumour resection is sufficient in NMIBC. In this context, therapy decisions for pT1G3 (pT1 high‐grade, hg) tumours are still challenging. pT1 hg comprises a problematic subgroup of tumours between the two main groups (NMIBC and MIBC). On the one hand, it has not invaded the muscle layer and is therefore accessible for endoscopic transurethral resection. On the other hand, at least a substantial part of these tumours has the ability for muscle invasion and early metastases. Decision‐making for or against cystectomy is currently based on parameters such as multifocality, recurrence rate (with or without instillation therapy) and assessment of the tumour by the surgeon, that is, at least in part, a ‘subjective’ evaluation. Until now, it has been almost impossible to define their invasive potential and, therefore, the risk of advanced disease of a pT1 hg tumour, although several efforts have been undertaken, including a histopathological substaging approach [1] and molecular subtyping based on a large‐scale gene expression analysis [2]. However, while these markers allow for a certain risk assessment, they are either hard to reproduce and standardise or are unsuitable for daily routine practice.

Hence, biomarkers that can easily characterise the invasive potential of tumours at a molecular level using standard laboratory methods with low turnover times could resolve these issues. In this regard, miRNAs represent a new and robust class of biomarkers that can be investigated in all sample types, including paraffin‐embedded tissues and body fluids. They are functionally involved in many signalling pathways that affect both physiological and pathological processes, such as tumorigenesis.

Our previous study identified four miRNAs (miR‐146b‐5p; miR‐155‐5p; miR‐138‐5p; and miR‐200a‐3p) that are differentially expressed between MIBC and NMIBC [3]. The major aim of the present study was to verify the diagnostic potential of these four miRNAs based on two independent cohorts and to develop a miRNA signature defining muscle invasiveness that was further applied to pT1 hg tumours. Firstly, we confirmed the previously observed expression differences using the two new cohorts. Secondly, we developed highly accurate classification models that could distinguish MIBC from pTa lg tumours using the investigated miRNAs. Additionally, we applied conformal prediction (CP) to our models. CP is a machine learning (ML) framework that guarantees that predictions are correct in a user‐defined percentage (here: 90% of cases) by eliminating incorrect model predictions, making the models more reliable and trustworthy for clinical applications. CP further improved our already highly accurate models by eliminating all remaining misclassifications. Lastly, the molecular subtypes were defined and correlated to miRNA expressions.

## 2 Materials and Methods

Two independent cohorts of selected MIBC and pTa low‐grade (pTa lg) cases were investigated retrospectively (cohort 1: Saarland University Hospital; cohort 2: Erlangen University Hospital) (Table S1). Informed consent was obtained from individuals, or data were analysed anonymously. MIBC tumour samples were obtained from either transurethral resection of bladder tumour (TURB) or cystectomy (CYS). In addition, 120 pT1 hg tumours from TURB were included. Informed consent was obtained from individuals, or data were analysed anonymously.

### 2.1 MiRNA Quantification

Tumour areas were dissected from five to 10 FFPE sections (7–10 μm). miRNA was isolated using the miRNeasy FFPE kit (Qiagen), according to the manufacturer's protocol. We performed quantitative real‐time polymerase chain reaction (qRT‐PCR) using TaqMan MicroRNA Reverse Transcription Kits (Applied Biosystems), followed by a polymerase chain reaction (PCR) step using specific TaqMan miRNA primers for miR‐138‐5p, miR‐146b‐5p, miR‐155‐5p and miR‐200a‐3p as well as miR‐191‐5p, miR‐361‐5p, and RNU48 as endogenous controls to normalise the miRNA input, and TaqMan Fast Advanced Master Mix (Applied Biosystems), according to the manufacturer's protocol. The qRT‐PCR was carried out in triplicate using LightCycler 480 (Roche Diagnostics Deutschland GmbH). Only miRNAs with a Ct < 35 were considered to be expressed. A *p*‐value < 0.05 was regarded as the significance threshold for upregulated or downregulated miRNA.

Statistical analyses were performed using GraphPad Prism software, Mann–Whitney and Kruskal–Wallis tests, and receiver operating characteristic (ROC) curves.

### 2.2 MIBC vs. pTa lg Classification

To distinguish MIBC from pTa lg, we trained classification models for each subset of the four investigated miRNAs using five ML algorithms: boosting trees, k‐nearest neighbours, random forests, support vector machines and vanilla neural networks. Two‐thirds of the larger cohort were used for training, and the smaller cohort was used for testing (the remaining third of the larger cohort was used to perform CP as described below). A fivefold cross‐validation was performed on the training data to find the hyperparameters that maximised the area under the ROC (receiver operating characteristic) curve for each model. All ML models were implemented in R using the packages ada [4], class [5], random‐Forest [6], kernlab [7], and nnet [5]. Details are provided in Supplementary Table S2.

For a given tumour sample, each ML model computes the probabilities of the sample belonging to the MIBC or pTa lg class. Both probabilities sum to 1, and the sample is predicted to belong to the class with the higher value. These probabilities can also be interpreted as a measure of how confident the model is in its prediction for the given sample: If both probabilities are close to 0.5, the classification is less certain than if one probability is considerably larger.

While class probabilities help to estimate model certainty, a guarantee of the correctness of individual predictions is strongly desirable, especially when ML models are applied in a clinical context. One sophisticated method to obtain such a guarantee is CP [8]. CP can extend ML models by altering their predictions so that they are guaranteed to be correct for unseen samples (i.e., test samples or samples in the routine clinical application of the model) in a user‐defined percentage of cases, usually 90%. CP requires a trained ML model and a so‐called *calibration dataset* that has the same format as the training and test data but is disjunct from both. The calibration dataset is used to determine a threshold for the predicted class probabilities, which is then used to alter predictions: If the class probability for one class (either MIBC or pTa lg) is greater than or equal to the threshold (i.e., the model is sufficiently confident in its prediction), the CP prediction consists of this single class and is equal to the prediction obtained without CP. In contrast, if the predicted probabilities are smaller than the threshold, CP predicts a set containing both classes {MIBC, pTa lg}, indicating that the given sample cannot be confidently assigned to either class.

We performed CP using the *adaptive prediction sets* algorithm by Angelopoulos and Bates [8], using one‐third of the training cohort as the calibration data.

### 2.3 Definition of Molecular Subtypes

To define molecular subtypes, spatially organised tissue microarrays of 1.5‐mm‐tissue cores (4 cores per tumour) were stained for CK5, CK14, CK20, GATA3, FOXA1, CD44 and UPK2 on a VENTANA BenchMark ULTRA autostainer (Ventana, Switzerland) according to a DAkkS (German accreditation society)‐accredited staining protocol and analysed using the immunoreactive score (IRS), as described previously [9], based on the recommendations for IHC‐based subtyping provided by the International Bladder Cancer Molecular Taxonomy Working Group [10]. The derived expression results were Z‐score normalised, and tumours were categorised as ‘luminal’ or ‘basal’ using an unsupervised Ward's linkage clustering algorithm with Euclidean distance as the metric scale. mRNA sequencing for consensus molecular subtyping was performed using the Lexogen QuantSeq 3′ mRNA‐Seq Kit FWD (Lexogen GmbH), as described previously [11]; gene counts were log2 transformed, and MIBC consensus subtype calling was performed in R v 4.1.0 using the single‐sample classifier R‐package BLCA subtyping v.2.1 (https://github.com/cit‐bioinfo/BLCAsubtyping).

## 3 Results

### 3.1 miRNA Expression in MIBC Compared to pTa lg Tumours

Using qRT‐PCR, the expressions of miR‐138‐5p, miR‐146b‐5p, miR‐155‐5p and miR‐200a‐3p, as well as three potential miRNA references for normalisation (miR‐191‐5p, miR‐361‐5p, RNU48), were quantified in the two cohorts (*n* = 66 and *n* = 183).

We decided to use miR‐361‐5p for normalisation, as it showed the most stable expression and did not differ between the MIBC and pTa lg groups.

**FIGURE 1:**
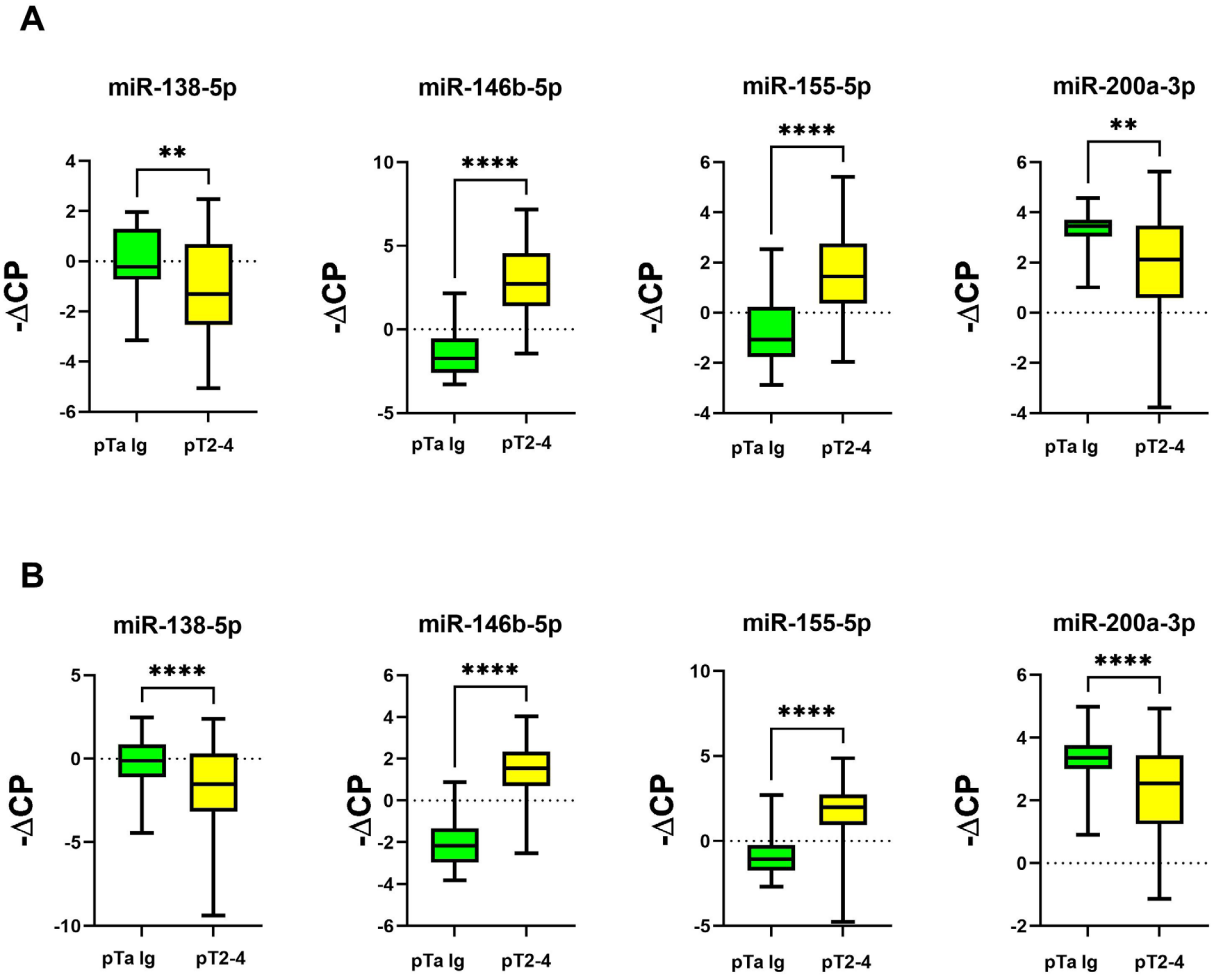
miRNA expression (normalised against miR‐361‐5p) in MIBC compared to pTa lg bladder cancer samples; A: Cohort 1; B: Cohort 2; (*): *p* ≤ 0.05; (**): *p* ≤ 0.01; (***): *p* ≤ 0.001; (****): *p* ≤ 0.0001.

**FIGURE 2:**
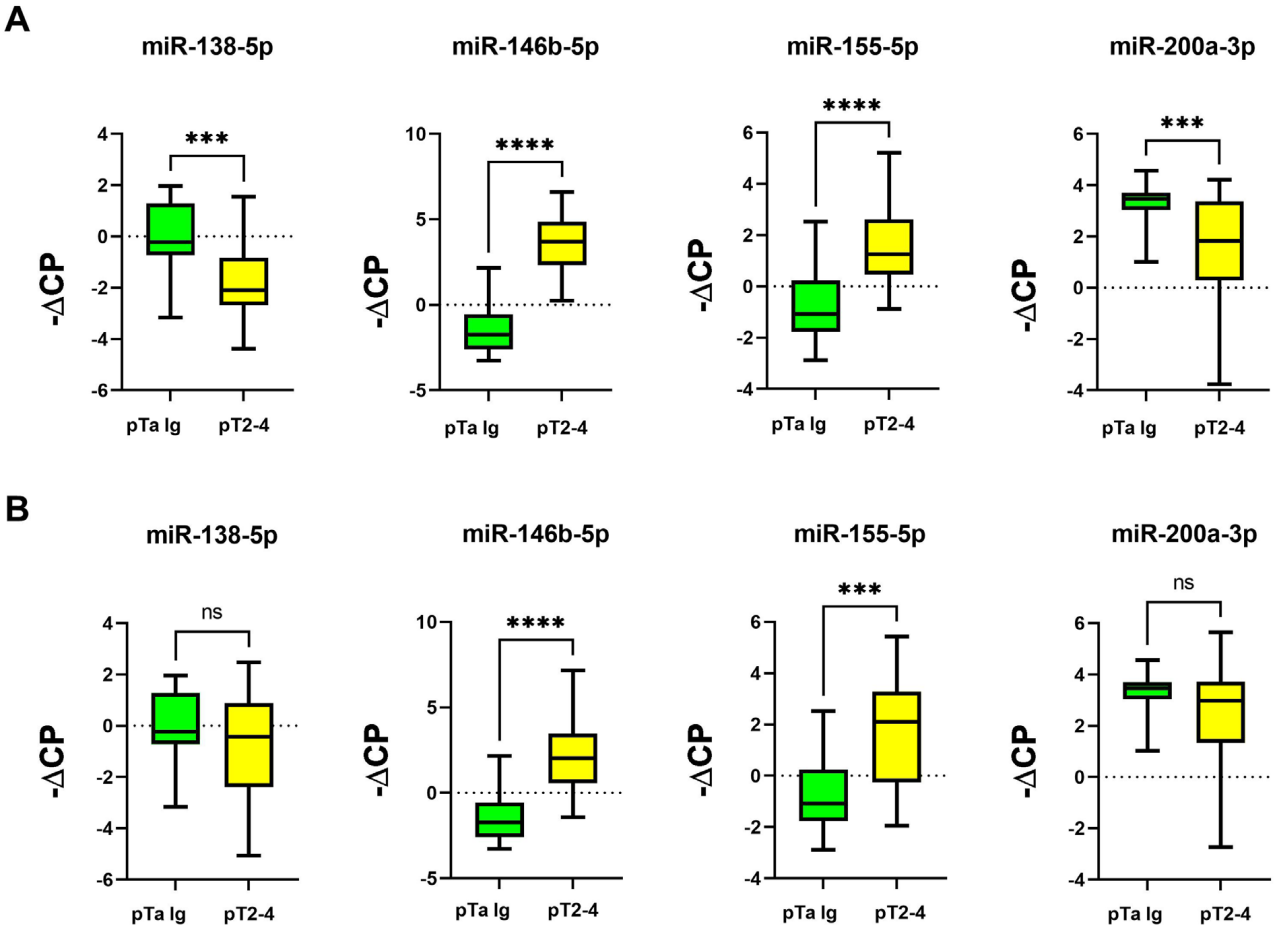
miRNA expression (normalised against miR‐361‐5p): Analysis of MIBC samples obtained from cystectomy (A) or from TURB (B) compared to pTa lg bladder cancer samples (cohort 1); (*): *p* ≤ 0.05; (**): *p* ≤ 0.01; (***): *p* ≤ 0.001; (****): *p* ≤ 0.0001.

**FIGURE 3:**
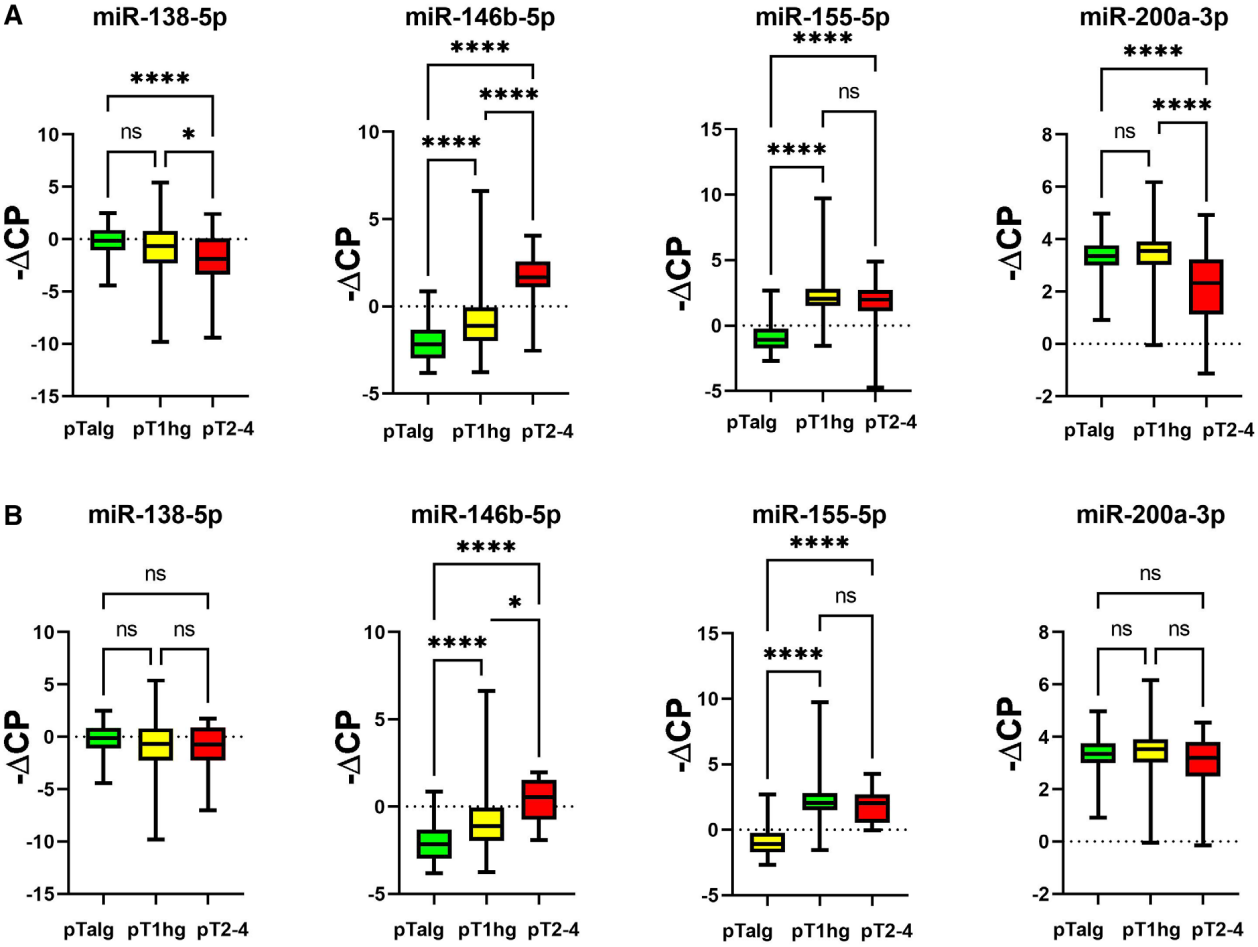
miRNA expression (normalised against miR‐361‐5p): Analysis of MIBC samples obtained from cystectomy (A) or from TURB (B), pT1 hg and pTa lg bladder cancer samples (cohort 2); (*): *p* ≤ 0.05; (**): *p* ≤ 0.01; (***): *p* ≤ 0.001; (****): *p* ≤ 0.0001.

In both cohorts, miR‐138‐5p and miR‐200a‐3p were significantly downregulated in MIBC compared to pTa lg (Figure 1; Table 1), while miR‐146b‐5p and miR‐155‐5p were significantly upregulated (Figure 1; Table 1). We then analysed MIBC samples from CYS and TURB separately in comparison with pTa lg. In both cohorts, significant expression differences between MIBC and pTa lg were confirmed for all four miRNAs when using the CYS MIBC samples and were also confirmed for miR‐146b‐5p and miR‐155‐5p when using the TURB MIBC samples (Figures 2 and 3; Table 1). However, no significant expression differences could be found for these two miRNAs between the TURB and CYS samples from MIBC in cohort 1 (Figure S1) which is in contrast to the findings for cohort 2 (miR‐146b‐5p, *p* = 0.0004; miR‐200a‐3p, *p* = 0.038, Figure S2). miRNA expression in MIBC in both cohorts did not differ between the pT categories.

**TABLE 1:** Statistical analysis of miRNA expression between non‐muscle‐invasive (NMIBC) and muscle‐invasive (MIBC) bladder cancer samples obtained after transurethral resection (TURB) or cystectomy (CYS) using Mann–Whitney‐*U* test.

Cohort	Comparison	miR‐138‐5p	miR‐146b‐5p	miR‐155‐5p	miR‐200a‐3p
1 (HOM)	pTa lg‐MIBC (TURB)	0.373	**< 0.0001**	**0.0002**	0.1216
	pTa lg‐MIBC (Cyst)	**0.0003**	**< 0.0001**	**< 0.0001**	**0.0006**
	pTa lg‐MIBC (all)	**0.004**	**< 0.0001**	**< 0.0001**	**0.002**
2 (ERL)	pTa lg‐MIBC (TURB)	0.2733	**< 0.0001**	**< 0.0001**	0.4309
	pTa lg‐MIBC (Cyst)	**< 0.0001**	**< 0.0001**	**< 0.0001**	**< 0.0001**
	pTa lg‐MIBC (all)	**< 0.0001**	**< 0.0001**	**< 0.0001**	**< 0.0001**

*Note:* statistically significant differences in bold.

Next, we performed ROC analyses (Table S3). Comparing the MIBC TURB to the pTa lg samples, a high AUC was reached for miR‐146b‐5p (cohort 1: 0.90; cohort 2: 0.91) and miR‐155‐5p (cohort 1: 0.83, cohort 2: 0.94) in both cohorts, in contrast to the observed results for miR‐138‐5p (cohort 1: 0.58, cohort 2: 0.59) and miR‐200a‐3p (cohort 1: 0.64, cohort 2: 0.56) (Figures S3 and S4). The AUC was larger when using MIBC CYS samples compared to MIBC TURB samples for all miRNAs in both cohorts (Figures S5 and S6). Accordingly, highly significant *p*‐values were achieved for all miRNAs when using the MIBC CYS samples and for miR‐146b‐5p and miR‐155‐5p when using MIBC TURB samples (Table 1).

### 3.2 Development of a Robust miRNA Signature Using Machine Learning Algorithms

**FIGURE 4:**
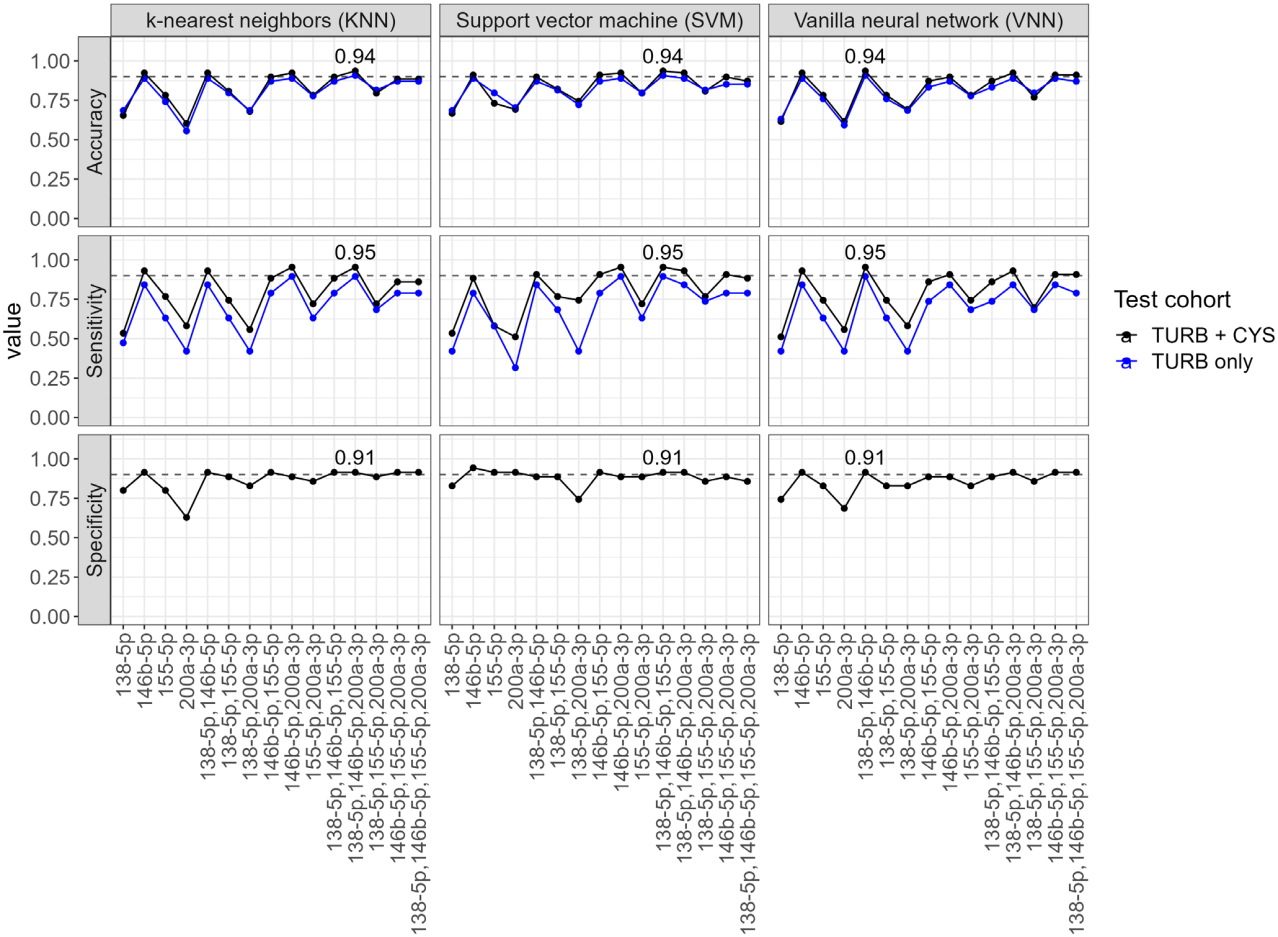
Classification performance: Accuracy, sensitivity (fraction of correctly classified MIBC samples) and specificity (fraction of correctly classified pTa lg samples) of classification models trained using different miRNA combinations (*x*‐axis) as input; black curves: complete test cohort (TURB and CYS samples); blue curves: TURB samples only; grey dashed horizontal lines: accuracy/sensitivity/specificity of 0.9. Specificity is equal for both curves, since the pTa lg group does not contain any CYS samples. Performance measures of the best‐performing models are denoted numerically. Results for random forests and boosting trees are provided in Figure S7.

**FIGURE 5:**
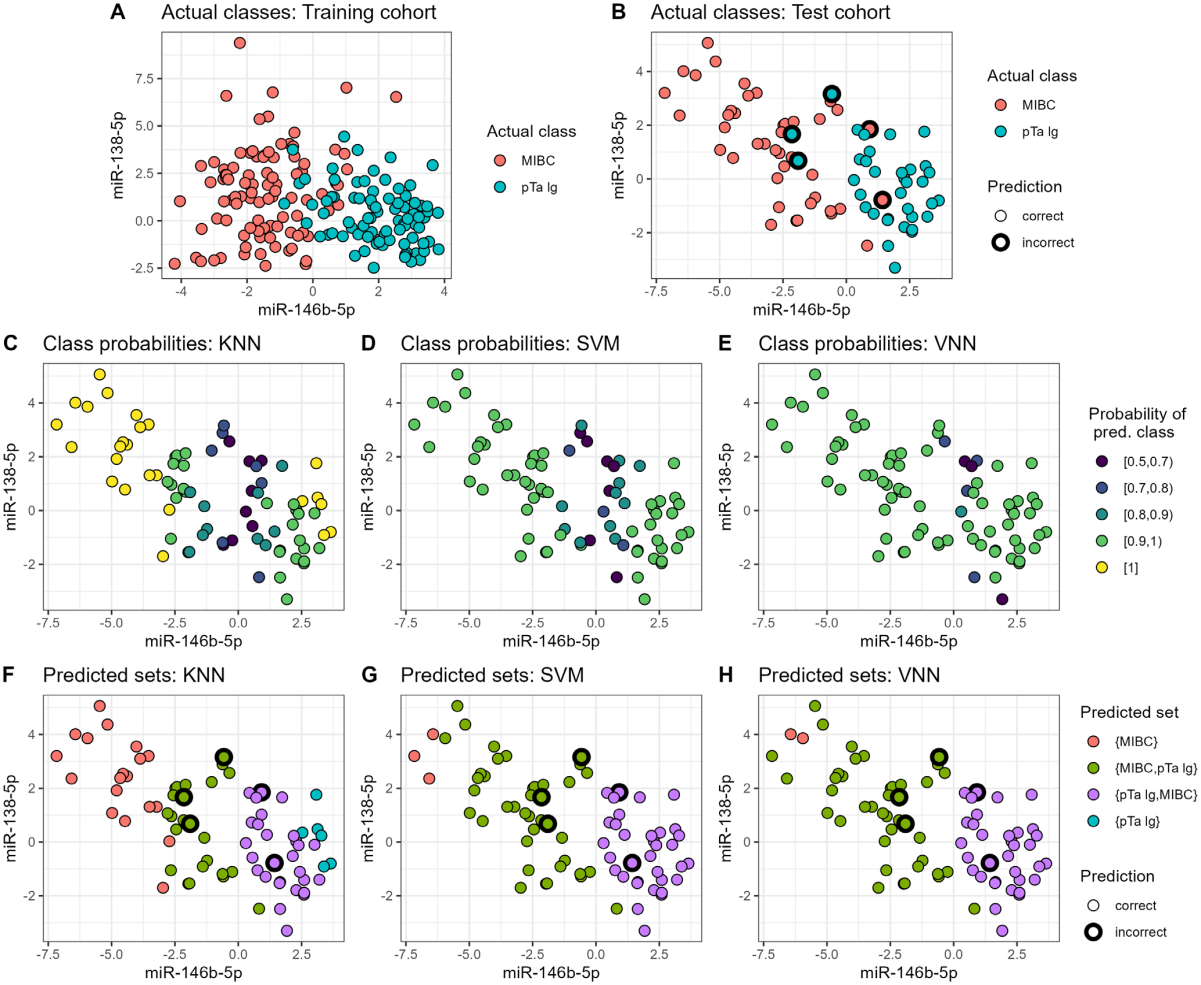
Predictions of machine learning models. (A and B) Expression of miR‐146b‐5p and miR‐138‐5p for the training and test cohorts, respectively. (B) Samples that were incorrectly predicted by the best‐performing KNN, SVM and VNN models are highlighted with a thick black outline. (C–D) Predicted class probabilities of the best KNN, SVM and VNN model, respectively; (E‐F) The prediction sets obtained by applying conformal prediction. For two‐class sets, the class with higher probability is listed first.

To determine which miRNA subset is most suited to distinguish MIBC from pTa lg, we trained classification models using five ML algorithms (Figure 4, Figure S7). The accuracy was above 0.85 for 40 of the 75 models. Noteworthily, models using only miR‐146b‐5p as a single predictor already reached an accuracy of up to 0.92. When model performance was evaluated using only the test cohort's TURB samples, sensitivity and accuracy slightly decreased. Specificity was unaffected since the pTa lg group does not contain any CYS samples. Three models achieved the best performance: a KNN model (*k* = 19, Table S2) with miR‐138‐5p, miR‐146b‐5p and miR‐200a‐3p as its features; a VNN model (size = 4, decay = 0.01) with miR‐138‐5p and miR‐146b‐5p as the features; and an SVM model (kernel = polynomial, C = 1, degree = 2, scale = 0.1) with miR‐138‐5p, miR‐146b‐5p and miR‐155‐5p as the features. All three models had an accuracy of 0.94 (0.91 for only TURB), a sensitivity of 0.95 (0.89 for only TURB) and a specificity of 0.91. Furthermore, all three models misclassified the same five samples (Figure 5B).

Based on their miR‐138‐5p and miR‐146b‐5p expression, the MIBC and pTa lg groups can be well separated, with some overlap where the expression value of miR‐146b‐5p approaches zero (Figure 5A,B). This is mirrored by the class probabilities derived from the KNN (Figure 5C), which decrease for samples closer to the centre of the point cloud while the SVM's and especially the VNN's probabilities are more uniform and thus less informative to distinguish the invasive potential of different samples (Figure 5D,E).

Next, we applied CP to our models (Figure 5F–H). For samples with relatively low prediction probabilities, which cannot be confidently classified as either MIBC or pTa lg, CP predicts two‐class sets (i.e., {MIBC, pTa lg}) to avoid misclassifications. Indeed, all five previously misclassified samples were predicted as two‐class sets by the KNN, SVM and VNN. Consequently, the predicted sets for the test cohort contained the true classes in all cases. However, the KNN yields more single‐class predictions than the SVM and VNN (32%, 4% and 3% of cases, respectively), where samples are confidently classified as either MIBC or pTa lg. Due to the more informative class probabilities and the increased number of single‐class CPs, the KNN is superior to the SVM and VNN, despite all models having the same accuracy.

### 3.3 Evaluation of miRNA Expression in TURB Samples

We then analysed pT1 hg tumours from TURB in comparison with pTa lg and MIBC from TURB (cohort 2). Expression of miR‐146b‐5p is between the two other groups and is significantly different from both, whereas expression of miR‐155‐5p is similar to MIBC and significantly different from pTa lg (Figure 3B). No significant differences were found for miR‐138‐5p and miR‐200a‐3p. When using MIBC samples from CYS, significant differences were found for miR‐138‐5p and miR‐200a‐3p, too (Figure 3A).

When applying the KNN model to the pT1 hg tumours, 32% of samples (cohort 2) are predicted as MIBC (Figure S8).

We further evaluated the classification of pT1 hg in TURB samples considering the final diagnosis in cystectomy samples (data available in 12 cases from cohort 1, Table S4). Five out of six cases with MIBC in final histology have been classified as MIBC by the KNN model. However, also five out of six cases with pTa/pTis in cystectomy have been predicted as MIBC, one with metachronous distant metastasis.

### 3.4 Definition of Molecular Subtypes

**FIGURE 6:**
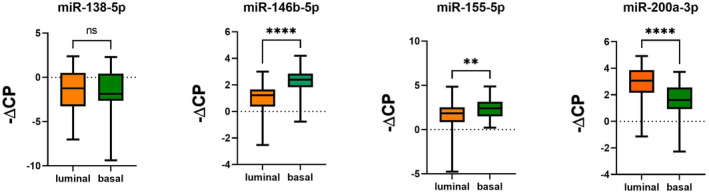
miRNA expression (normalised against miR‐361‐5p) in MIBC (cohort 2); comparison between luminal and basal subtypes defined by immunohistochemistry; (*): *p* ≤ 0.05; (**): *p* ≤ 0.01; (***): *p* ≤ 0.001; (****): *p* ≤ 0.0001.

Furthermore, the molecular subtypes in MIBC of cohort 2 were defined. Using immunochemistry, the luminal and basal subtypes were found in 37.5% and 72.5% of the samples, respectively. miR‐146b‐5p and miR‐155‐5p were significantly more highly expressed in basal MIBC, whereas miR‐200a‐3p was expressed significantly less in basal MIBC (Figure 6). miR‐138‐5p expression did not differ significantly between the subtypes. Compared to pTa lg, miR‐138‐5p, miR‐146‐5p and miR‐155‐5p were significantly differentially expressed in both luminal and basal MIBC, in contrast to miR‐200a‐3p, which was less highly expressed only in basal MIBC (Figure S9). Using gene expression analysis, all six subtypes were identified: luminal papillary (LumP) in 9 (9.5%), luminal unstable (LumU) in 9 (9.5%), basal/squamous (Bas/Sq) in 42 (44.2%), stroma‐rich in 33 (34.7%) and neuroendocrine‐like (NeLike) in two samples (2.1%). Luminal‐non‐specified was found in only one case and was excluded from the statistics. Results were similar to those described above (Figure S10).

## 4 Discussion

Our results confirm the large potential of miRNAs being used as biomarkers for differentiation between pTa lg NMIBC and MIBC. Furthermore, our data strongly support the hypothesis that distinct pathways with distinct molecular alterations characterise the development of papillary low grade and MIBC [12]. Since our overarching goal is to develop miRNA models that can estimate the risk of muscle invasion before performing a potentially avoidable cystectomy, our estimates need to be accurate even when only TURB samples are considered. Therefore, we analysed MIBC samples from cystectomies and TURB separately; we validated the four miRNAs from our previous study using the two independent cohorts' cystectomy MIBC samples and found that miR‐146b‐5p and miR‐155 were still significantly differentially expressed in TURB samples. Considering the very high discriminatory power of these two miRNAs reflected in the ROC analyses, our data support the hypothesis that the diagnosis of muscle invasiveness is possible using samples obtained from TURB. However, this has to be evaluated in larger cohorts using only TURB samples. On the one hand, the minor differences observed between TURB and cystectomy samples for two miRNAs might be based on the smaller number of TURB samples. On the other hand, it seems possible that the deeper and more invasive parts of the MIBC that are analysed from cystectomy samples have a higher probability of representing an invasive miRNA signature.

As tumour progression, including invasion, is characterised by complex molecular alterations, as shown for the four miRNAs, it is important to define miRNA signatures using ML algorithms to create robust models instead of using single miRNAs. The trained ML models had high accuracy, even when only tested on TURB samples, indicating that the investigated miRNAs are suitable biomarkers for distinguishing MIBC from pTa lg NMIBC. Since the models were trained and tested on different cohorts, the predictions seem robust for different data sources.

Even though miR‐155‐5p effectively distinguished pTa lg from MIBC in the previous ROC analyses, it is not included in two of the three best‐performing models. This might be explained by the relatively high expression correlation of 0.62 between miR‐155‐5p and miR‐146b‐5p. Consequently, both miRNAs might provide similar information, but since miR‐146b‐5p alone yields more accurate predictions than miR‐155‐5p alone, only miR‐146b‐5p is included in the best KNN and VNN models. In contrast, miR‐138‐5p and miR‐200a‐3p are less predictive on their own but seem helpful to classify cases where miR‐146b‐5p alone does not suffice.

We additionally investigated the prediction probabilities derived from the best‐performing KNN, SVM and VNN models. These probabilities denote how confident a model is in its prediction for a given sample. The probabilities obtained from the KNN vary considerably, indicating that some samples are easier to classify than others. Furthermore, the KNN probabilities for misclassified samples are comparatively low, which is desirable, as they indicate the model's uncertainty in classifying these cases.

Lastly, we applied CP to our models, a method that predicts a sample as being either MIBC or pTa lg only when the corresponding class probability is sufficiently large. CP guarantees that the actual class of a sample should be contained within the CP predictions for a user‐specified percentage (here: 90%) of cases. This *certainty guarantee* is highly desirable for the clinical application of ML models: First, misclassifications are avoided because the model does not have to decide on a single class in uncertain cases. Second, the obtained single‐class predictions are more trustworthy because they represent cases where the model was sufficiently confident in its decision. By applying CP to our models, all misclassifications in the test cohort were eliminated. Compared to the SVM and VNN, the KNN confidently assigned a single class to a sample much more frequently, thus yielding more informative predictions. Consequently, the KNN is superior for predicting muscle invasion despite having the same accuracy as the other two models.

However, several samples could not be assigned to a single class when using CP. This should not necessarily be interpreted as a fault of CP, but rather as an indication that, based on the data and models at hand, only a subset of samples can be confidently classified. In such cases, the benefit of the model is less a specific prediction that might affirm or question the opinion of a human expert, but rather an indication that the sample at hand might require careful investigation. Overall, we believe that CP could create trust in the application of ML for medical decision support by ensuring highly accurate predictions while also indicating when a sample cannot be classified confidently.

Based on our results, the miRNA signature of a tumour could be helpful in defining the invasive potential of cases with a questionable histology l, especially in pT1 hg tumours after TURB. We excluded theses tumours from the NMIBC class as they represent a more heterogeneous group concerning prognosis, on the one hand, and a distinct histological group without invasion in the muscle layer but with poor differentiation, on the other hand. When analysing pT1 hg tumours separately, the miRNA expression overlapped with both pTa lg and MIBC for three miRNAs. In contrast, the expression of miR‐155‐5p is at the same level as in MIBC and completely different from pTa lg NMIBC. Therefore, it seems that this miRNA characterises the invasive potential of BC in general and not concerning the muscle layer. When applying the KNN model to the pT1 hg samples, predictions were heterogeneous, underlining that some pT1 hg cases resemble pTa lg and others resemble MIBC. In a small cohort, follow‐up data were available. In almost all cases in which cystectomy was performed, muscle invasiveness was predicted in TURB samples using the KNN model. As in six out of 12 cases, NMIBC was still found in final histology; one could interpret this as false‐positive results and, therefore, low specificity. However, it is also possible, and in our view more likely, that the tumour was resected at a time point when the tumour cells had not yet reached the muscle layer. As in all these cases, the decision for an early cystectomy is based on multiple individual factors such as multifocality, macroscopic features observed by the surgeon during TURB, recurrence dynamics, or BCG failure. Our classification model supports the evaluation of these cases as aggressive tumours. Of course, the data have to be evaluated in larger cohorts with longer follow‐up data to further study these assumptions.

Several publications confirmed miRNAs as biomarkers in cell‐free supernatant or sediments derived from urine samples from BC patients [13]. In our previous study, the differential expression of miR‐146b‐5p and miR‐155‐5p in MIBC compared to NMIBC was verified in urinary extracellular vesicles for the first time [3]. Future studies on larger cohorts must investigate whether the invasive potential of tumours can be accurately predicted based on a miRNA analysis of urine.

The molecular subtypes of MIBC have been proven to be associated with prognosis and therapy response [9, 14, 15]. miR‐146b‐5p, miR‐155‐5p and miR‐200a‐3p are differentially expressed between basal and luminal subtypes, supporting data obtained from a comprehensive molecular characterisation of the TCGA cohort [15]. Taken together, these three miRNAs characterise not only invasiveness but also the more aggressive basal subtype.

The four previously identified miRNAs have been investigated as biomarkers in relation to their functional role in the tumour‐related processes in multiple tumour entities, particularly in BC. miR‐138‐5p is known as a tumour suppressor and is known to be downregulated in several tumour types [16]. Awadalla et al. found a significantly lower expression of miR‐138‐5p in MIBC compared to pT1 tumours, confirming our results, and discussed HIF1a as a target, as it is more highly expressed in MIBC [17] and seems to be strongly inversely correlated with TERT, PD‐L1 and PD‐L2, as well as survivin expression [18, 19].

A higher expression of miR‐146b‐5p in the advanced stages of BC, as well as its association with M2 macrophage infiltration, has been reported [20]. miR‐146b‐5p seems to be a target of long non‐coding RNA lnc‐STYK1‐2, and it promotes proliferation, migration and invasion via ITGA2 as well as AKT/STAT3/NF‐κB signalling [21]. Depending on the tumour type, this miRNA can act as a tumour promoter [22, 23, 24, 25] or suppressor [26, 27].

A correlation was found between a higher expression of miR‐155 and higher BC stages, as well as shorter progression‐free survival [28]. Therefore, it seems that miR‐155 is associated with an invasive phenotype, as confirmed by in vitro experiments demonstrating that miR‐155 increases both proliferation and invasion [29]. Several other studies have found overexpression of miR‐155 in the tumour tissue of BC but did not investigate its possible association with invasiveness.

Many publications have proven miR‐200a‐3p to be a tumour suppressor. Its downregulation is associated with proliferation, EMT, invasion, metastasis and poor prognosis in different tumour types [30, 31, 32, 33]. A lower expression was found in tumour tissues and in urine, especially in advanced tumour stages of BC [3, 34], although some contradictory results have been reported [35]. To understand the role and function of a given miRNA in BC, it seems important to investigate NMIBC and MIBC separately.

Our study has some limitations. The cohorts have been retrospectively selected and analysed. Furthermore, the number of MIBC samples from TURB is small and the model has to be validated in a larger TURB cohort. Follow‐up data on pT1 hg tumours were available only from a very small number of patients, especially from those without early cystectomy and other treatment such as BCG instillation could not be evaluated.

In conclusion, our data further confirms distinct molecular pathways of non‐invasive low grade NMIBC and MIBC. By using machine learning algorithms, we defined a robust 3‐miRNA signature that differentiates MIBC from pTa lg with high accuracy even in TURB samples. After prospective validation in independent cohorts including pT1 hg tumours, it might be applied in routine diagnostics to define the invasive potential of a tumour in addition to its histopathological examination and therefore support the clinical decisions that have to be made by the patient and clinician, namely to choose between performing radical a cystectomy and pursuing bladder‐conserving strategies. This could be especially helpful for therapeutic decision‐making in T1 hg tumours. Furthermore, developing non‐invasive diagnostic methods that use urine samples seems possible with this miRNA panel.

## Author Contributions

**Lea Eckhart:** data curation (equal), formal analysis (equal), investigation (equal), writing – review and editing (equal). **Sabrina Rau:** data curation (equal), formal analysis (equal), investigation (equal). **Markus Eckstein:** data curation (equal), formal analysis (equal), investigation (equal), writing – original draft (equal). **Phillip R. Stahl:** resources (equal), writing – review and editing (equal). **Hiresh Ayoubian:** methodology (equal), writing – review and editing (equal). **Julia Heinzelbecker:** resources (equal), writing – review and editing (equal). **Farzaneh Zohari:** writing – review and editing (equal). **Arndt Hartmann:** methodology (equal), writing – review and editing (equal). **Michael Stöckle:** conceptualization (equal), writing – review and editing (equal). **Hans‐Peter Lenhof:** data curation (equal), methodology (equal), project administration (equal), writing – review and editing (equal). **Kerstin Junker:** conceptualization (equal).

## Ethics Statement

The research protocol was approved by an institutional review board.

Consent: All informed consent was obtained from the subject(s) and/or guardian(s).

Registry and the Registration No. of the Study/Trial: The authors have nothing to report.

Animal Studies: The authors have nothing to report.

## Conflicts of Interest

The authors declare no conflicts of interest.

## Supporting information


**Figure S1.** miRNA expression (normalised against miR‐361‐5p) in MIBC samples obtained from cystectomy compared to MIBC samples obtained from TURB (cohort 1); (*): *p* ≤ 0.05; (**): *p* ≤ 0.01; (***): *p* ≤ 0.001; (****): *p* ≤ 0.0001.

**Figure S2.** miRNA expression (normalised against miR‐361‐5p) in MIBC samples obtained from cystectomy compared to MIBC samples obtained from TURB (cohort 2); (*): *p* ≤ 0.05; (**): *p* ≤ 0.01; (***): *p* ≤ 0.001; (****): *p* ≤ 0.0001.

**Figure S3.** ROC curve analysis to distinguish MIBC from pTa lg tumours using TURB MIBC samples (cohort 1).

**Figure S4.** ROC curve analysis to distinguish MIBC from pTa lg tumours using TURB MIBC samples (cohort 2).

**Figure S5.** ROC curve analysis to distinguish MIBC from pTa lg tumours using cystectomy MIBC samples (cohort 1).

**Figure S6.** ROC curve analysis to distinguish MIBC from pTa lg tumours using cystectomy MIBC samples (cohort 2).

**Figure S7.** Classification performance of further ML models: accuracy, sensitivity (fraction of correctly classified MIBC samples), and specificity (fraction of correctly classified pTa lg samples) of boosting trees and random forests trained using different miRNA combinations (*x*‐axis) as input; black curves: complete test cohort (TURB and CYS samples); blue curves: TURB samples only; grey dashed horizontal lines: accuracy/sensitivity/specificity of 0.9.

**Figure S8.** miRNA expression (normalised against miR‐361‐5p) and KNN predictions of 108 pT1 hg cases (cohort 2).

**Figure S9.** miRNA expression (normalised against miR‐361‐5p) in MIBC (cohort 2); comparison of luminal and basal subtypes with pTa lg; defined by immunohistochemistry; (*): *p* ≤ 0.05; (**): *p* ≤ 0.01; (***): *p* ≤ 0.001; (****): *p* ≤ 0.0001.

**Figure S10.** miRNA expression (normalised against miR‐361‐5p) in MIBC (cohort 2); comparison between molecular subtypes defined by RNA sequencing; (*): *p* ≤ 0.05; (**): *p* ≤ 0.01; (***): *p* ≤ 0.001; (****): *p* ≤ 0.0001.

**Table S1.** Cohort characteristics.

**Table S2.** Overview of machine learning (ML) algorithms.

**Table S3.** Results obtained by receiver operating characteristic (ROC) curve statistics; AUC, area under the curve.

**Table S4.** Prediction of muscle invasiveness in TURB pT1 hg tumour samples using the KNN model and conformal prediction (CP) and final histology in cystectomy (CYS) tumour samples.

## Data Availability

Data available on request from the authors.
