# Supplementary figures and images for: Machine Learning Accurately Predicts Muscle Invasion of Bladder Cancer Based on Three miRNAs
Source: J Cell Mol Med. 2025 Feb 10;29(3):e70361. doi: 10.1111/jcmm.70361 (PMC11810526; doi:10.1111/jcmm.70361)

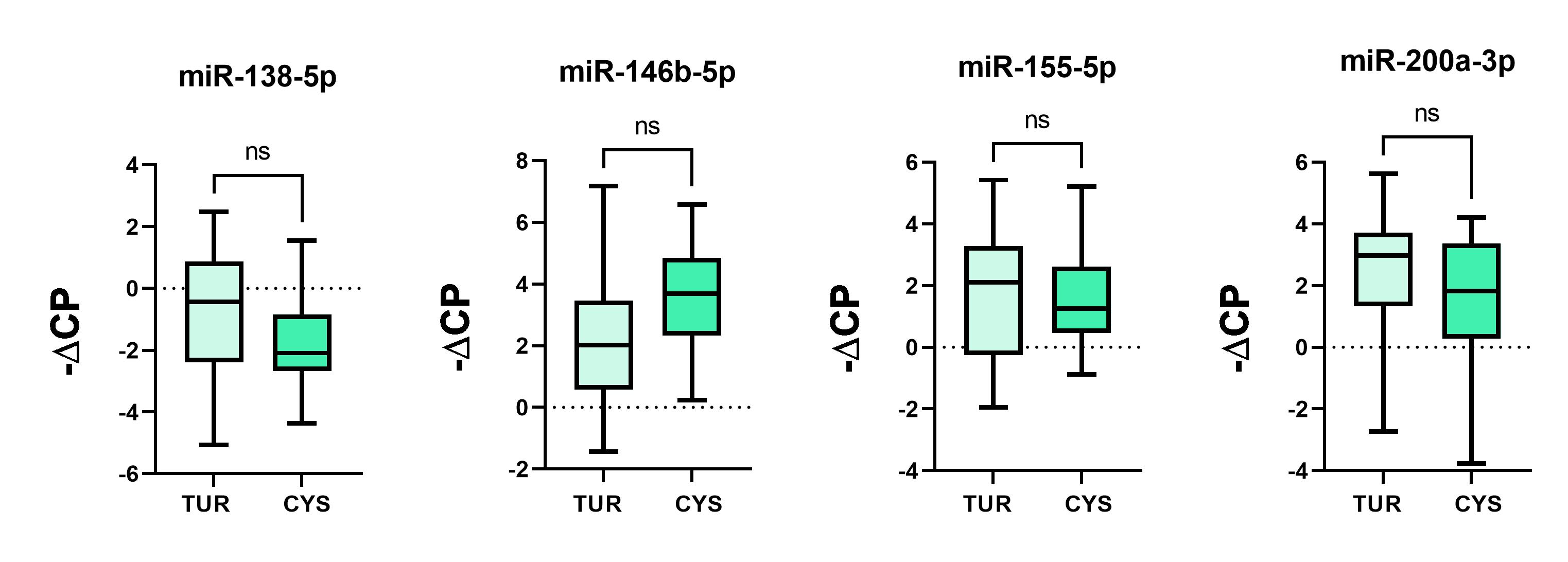

Supplement: Supplementary file 1 — Figure S1. miRNA expression (normalised against miR‐361‐5p) in MIBC samples obtained from cystectomy compared to MIBC samples obtained from TURB (cohort 1); (*): p ≤ 0.05; (**): p ≤ 0.01; (***): p ≤ 0.001; (****): p ≤ 0.0001. [file JCMM-29-e70361-s008.jpg]

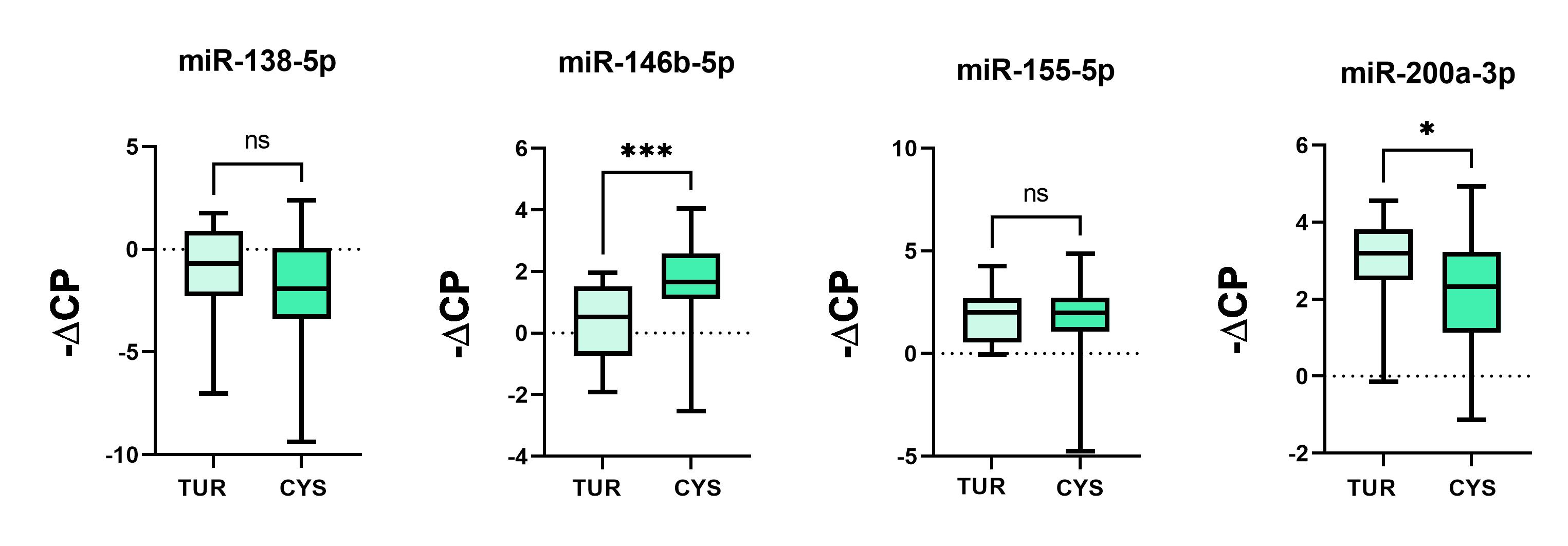

Supplement: Supplementary file 2 — Figure S2. miRNA expression (normalised against miR‐361‐5p) in MIBC samples obtained from cystectomy compared to MIBC samples obtained from TURB (cohort 2); (*): p ≤ 0.05; (**): p ≤ 0.01; (***): p ≤ 0.001; (****): p ≤ 0.0001. [file JCMM-29-e70361-s005.jpg]

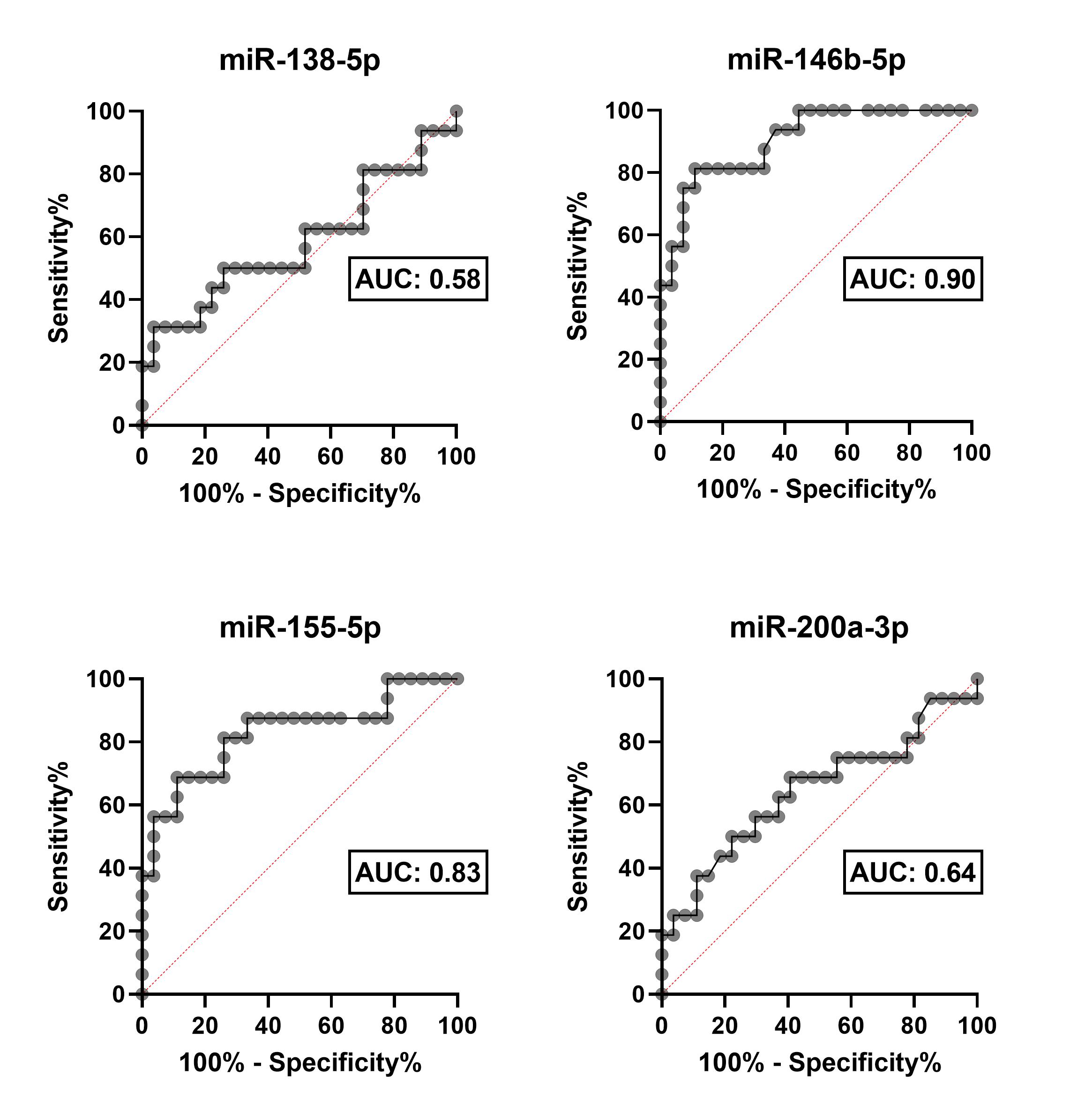

Supplement: Supplementary file 3 — Figure S3. ROC curve analysis to distinguish MIBC from pTa lg tumours using TURB MIBC samples (cohort 1). [file JCMM-29-e70361-s012.jpg]

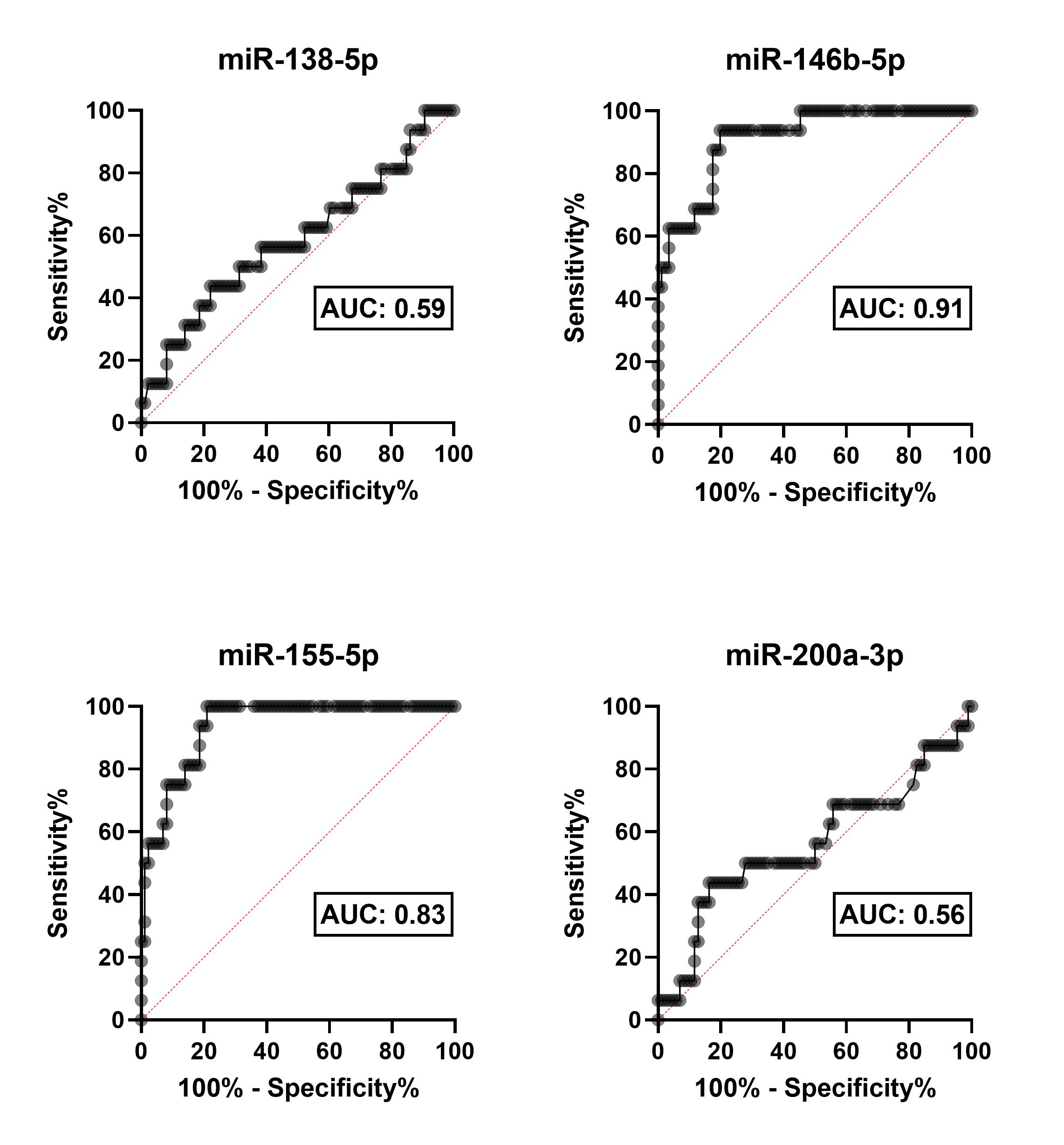

Supplement: Supplementary file 4 — Figure S4. ROC curve analysis to distinguish MIBC from pTa lg tumours using TURB MIBC samples (cohort 2). [file JCMM-29-e70361-s009.jpg]

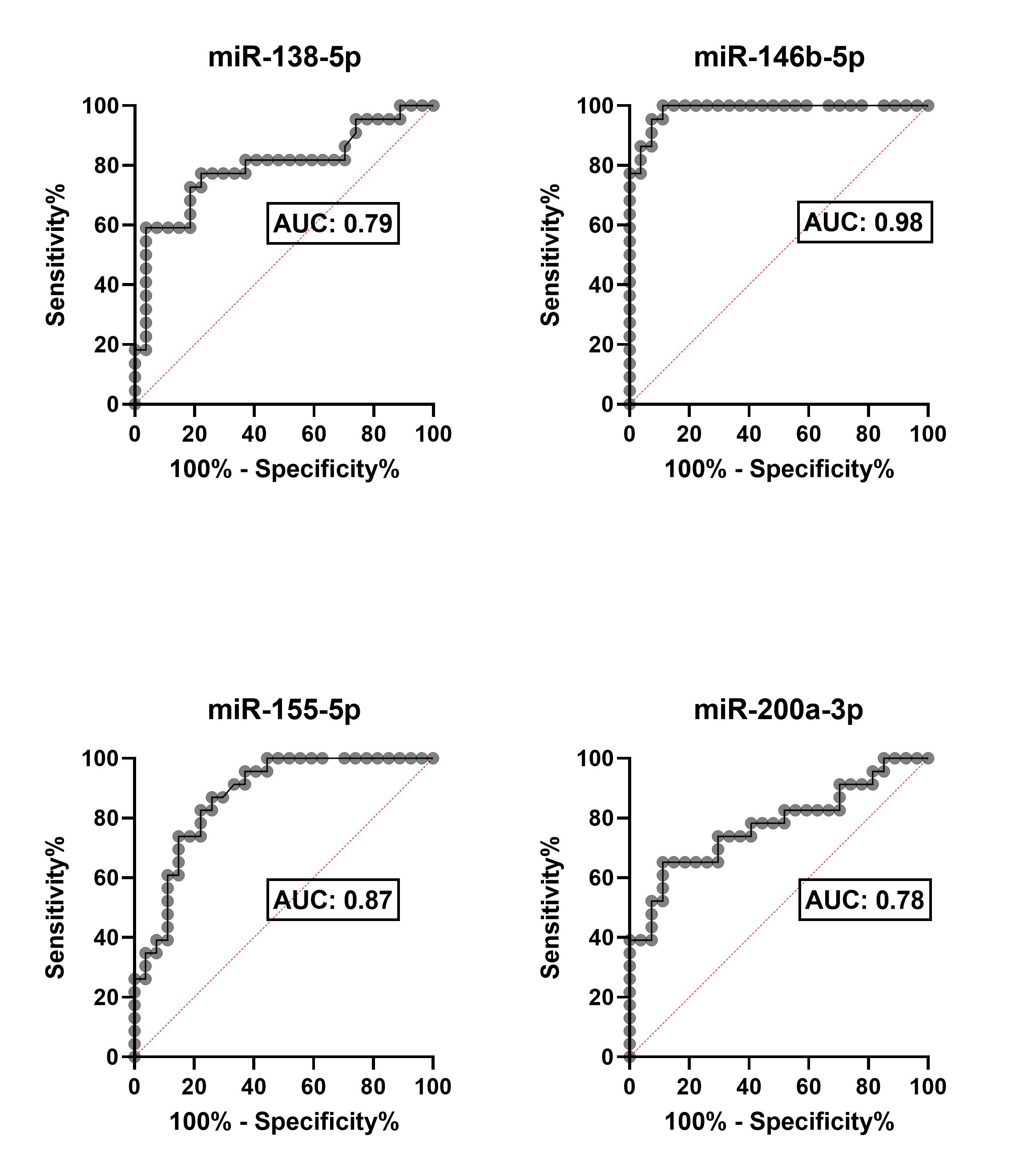

Supplement: Supplementary file 5 — Figure S5. ROC curve analysis to distinguish MIBC from pTa lg tumours using cystectomy MIBC samples (cohort 1). [file JCMM-29-e70361-s003.jpg]

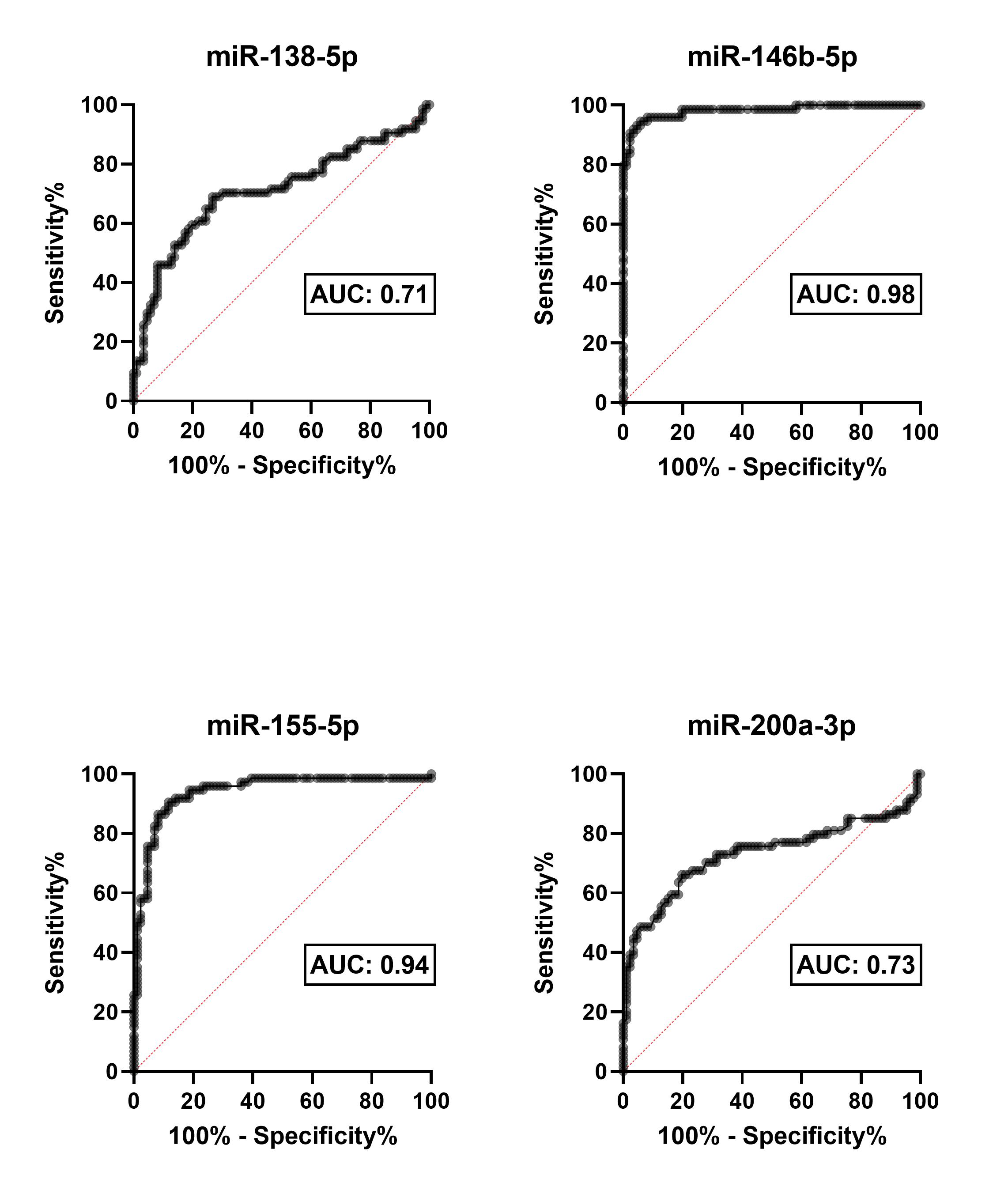

Supplement: Supplementary file 6 — Figure S6. ROC curve analysis to distinguish MIBC from pTa lg tumours using cystectomy MIBC samples (cohort 2). [file JCMM-29-e70361-s002.jpg]

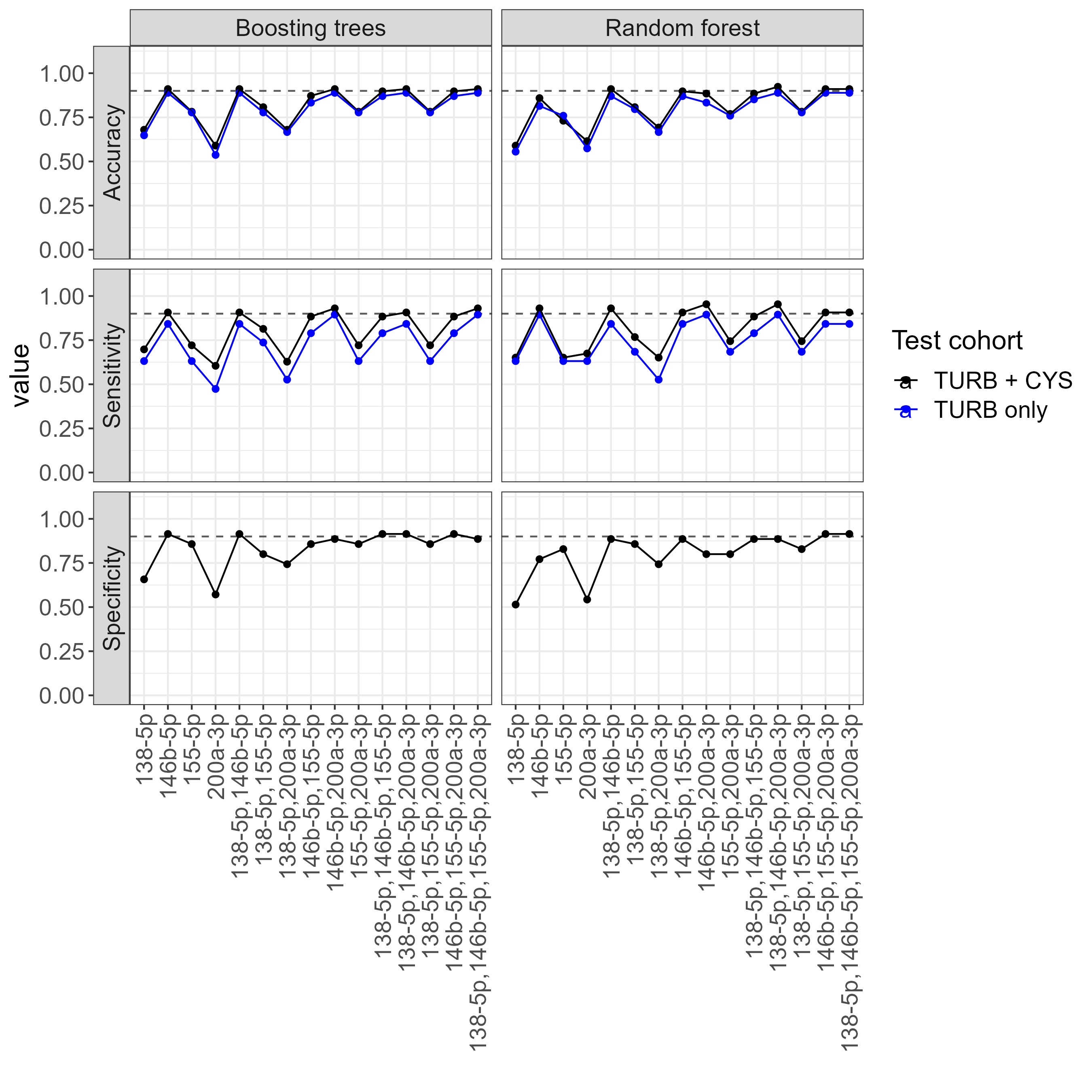

Supplement: Supplementary file 7 — Figure S7. Classification performance of further ML models: accuracy, sensitivity (fraction of correctly classified MIBC samples), and specificity (fraction of correctly classified pTa lg samples) of boosting trees and random forests trained using different miRNA combinations (x‐axis) as input; black curves: complete test cohort (TURB and CYS samples); blue curves: TURB samples only; grey dashed horizontal lines: accuracy/sensitivity/specificity of 0.9. [file JCMM-29-e70361-s010.png]

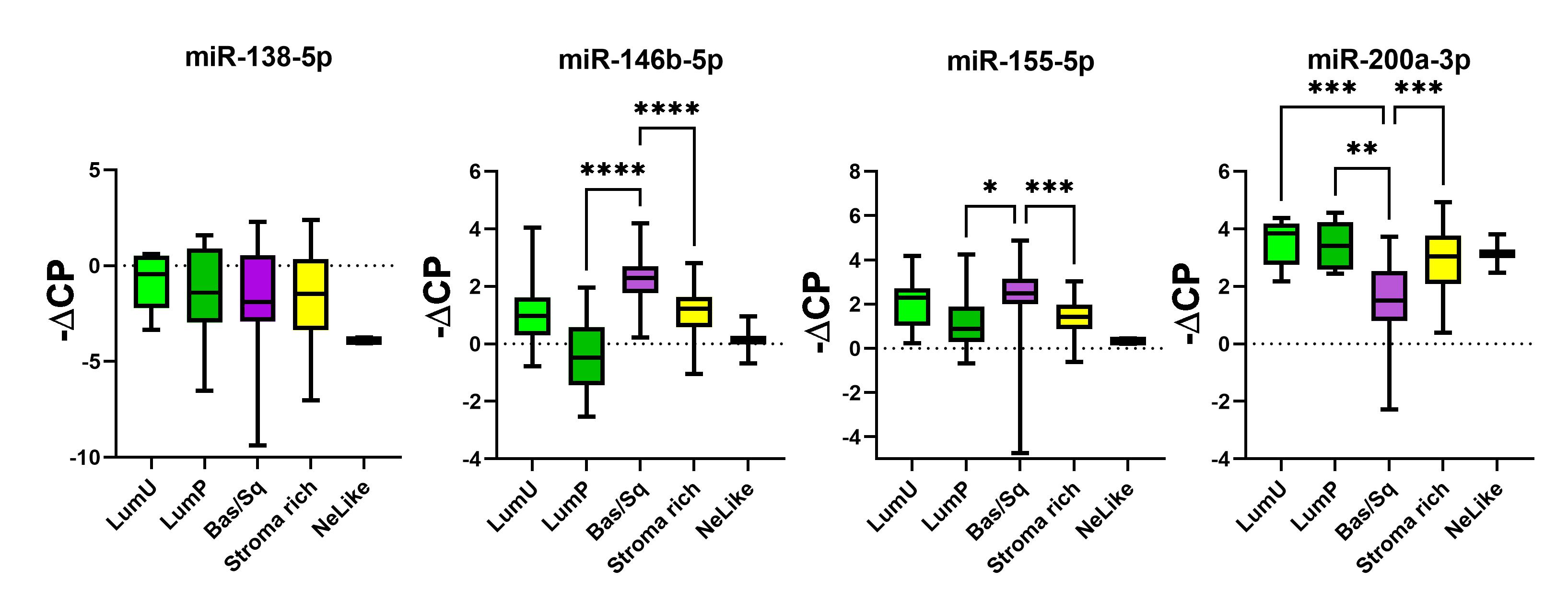

Supplement: Supplementary file 8 — Figure S8. miRNA expression (normalised against miR‐361‐5p) and KNN predictions of 108 pT1 hg cases (cohort 2). [file JCMM-29-e70361-s013.jpg]

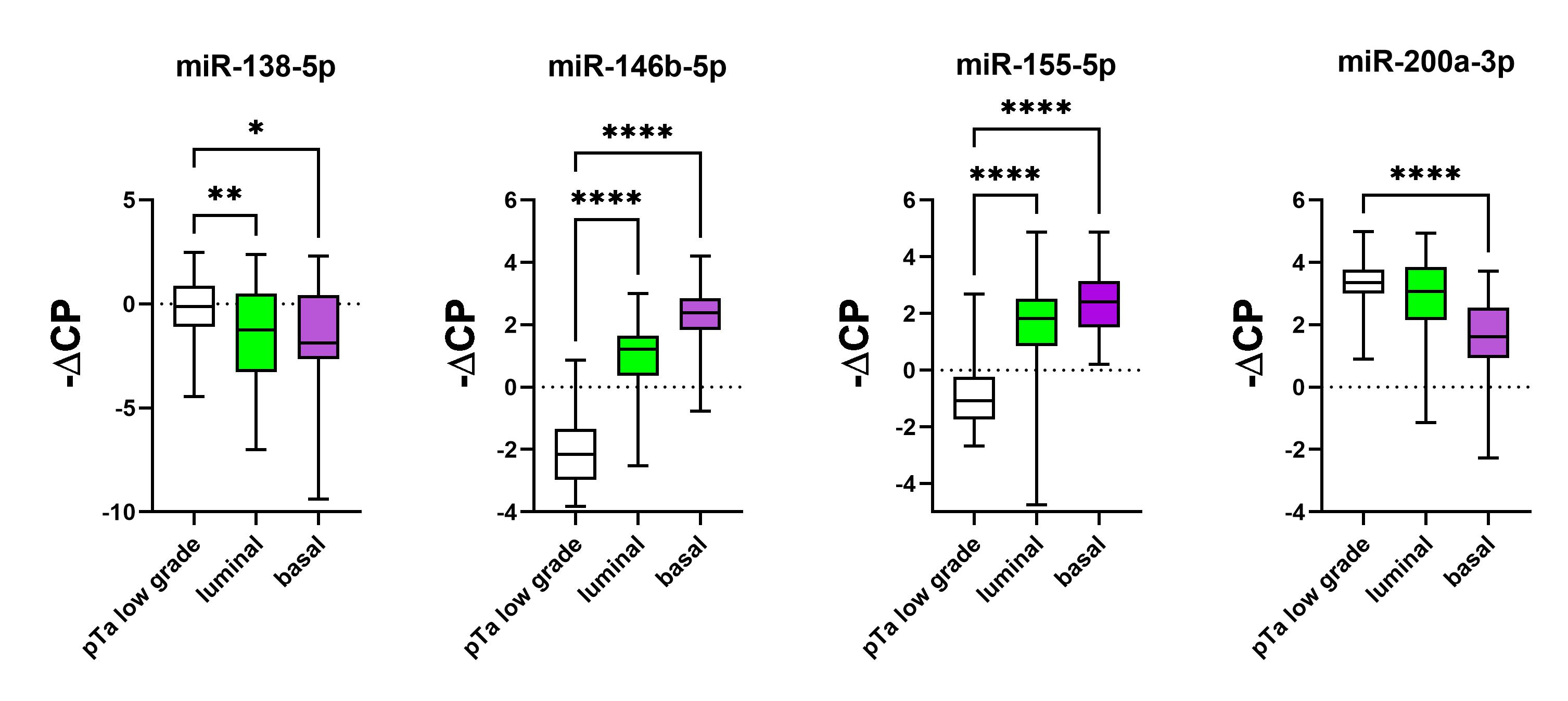

Supplement: Supplementary file 9 — Figure S9. miRNA expression (normalised against miR‐361‐5p) in MIBC (cohort 2); comparison of luminal and basal subtypes with pTa lg; defined by immunohistochemistry; (*): p ≤ 0.05; (**): p ≤ 0.01; (***): p ≤ 0.001; (****): p ≤ 0.0001. [file JCMM-29-e70361-s006.jpg]

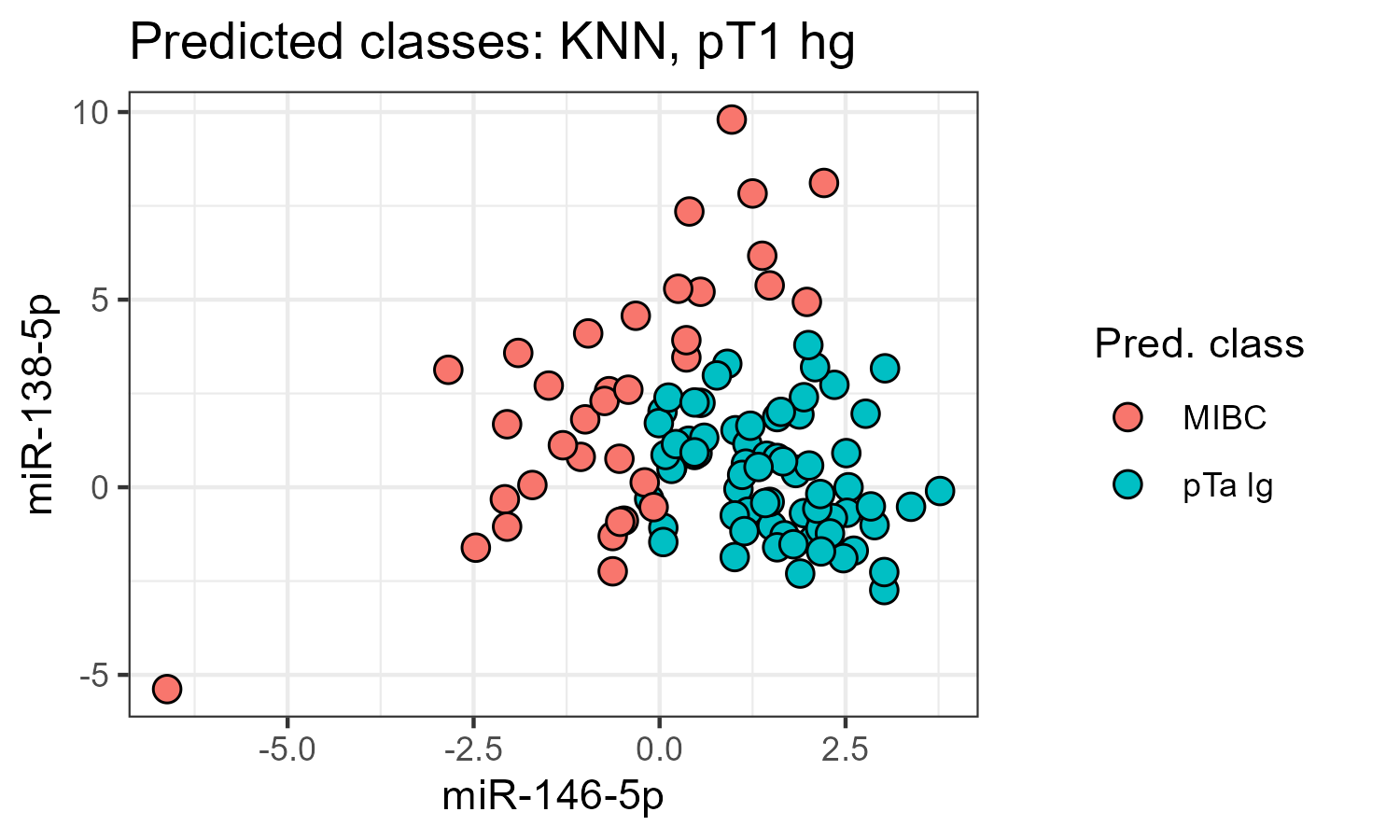

Supplement: Supplementary file 10 — Figure S10. miRNA expression (normalised against miR‐361‐5p) in MIBC (cohort 2); comparison between molecular subtypes defined by RNA sequencing; (*): p ≤ 0.05; (**): p ≤ 0.01; (***): p ≤ 0.001; (****): p ≤ 0.0001. [file JCMM-29-e70361-s011.png]
