# Supplementary material for: Machine Learning Accurately Predicts Muscle Invasion of Bladder Cancer Based on Three miRNAs
Source: J Cell Mol Med. 2025 Feb 10;29(3):e70361. doi: 10.1111/jcmm.70361 (PMC11810526; doi:10.1111/jcmm.70361)
Supplement: Supplementary file 11 — Table S1. Cohort characteristics. [file JCMM-29-e70361-s007.docx]

|  | **Cohort 1 (HOM) n (%)** | **Cohort 2 (ERL) n (%)** | **all** |
| --- | --- | --- | --- |
| **pTa lg (NMIBC)** | 27 | 86 | 113 |
| **pT2-pT4 hg (MIBC)** | 39 | 97 | 136 |
| pT2 | 23 (59.0) | 30 (30.9) | 53 |
| pT3 | 12 (30.8) | 45 (46.4) | 57 |
| pT4 | 4 (10.2) | 22 (22.7) | 26 |
| pT2-pT4 hg (MIBC) TURB | 16 | 23 | 39 |
| pT2-pT4 hg (MIBC) CYS | 23 | 74 | 97 |
| **pT1 hg** | 12 | 108 | 120 |

Suppl. Table S1: Cohort characteristics
