# Supplementary material for: Machine Learning Accurately Predicts Muscle Invasion of Bladder Cancer Based on Three miRNAs
Source: J Cell Mol Med. 2025 Feb 10;29(3):e70361. doi: 10.1111/jcmm.70361 (PMC11810526; doi:10.1111/jcmm.70361)
Supplement: Supplementary file 12 — Table S2. Overview of machine learning (ML) algorithms. [file JCMM-29-e70361-s004.docx]

| Algorithm | R Package | Parameter | Tuned Values |
| --- | --- | --- | --- |
| Boosting trees | ada  (v. 2.0.5) [32] | *iter* (number of trees)  *maxdepth* (max. tree depth)  *nu* (regularization factor for predictions of individual trees) | 1 – 5  5 – 25, increment 5  0.1, 1 |
| K-nearest neighbors | class  (v. 7.3.19) [33] | *k* (number of neighboring samples considered for class prediction) | 1 – 25, increment 2 |
| Random forest | random Forest (v. 4.7.1.1) [34] | *mtry* (number of randomly selected features for each split) | 1 – 4 |
| Vanilla neural network | nnet  (v. 7.3.16) [33] | *size* (number of nodes in hidden layer)  *decay* (factor for weight regularization) | 1 – 10 0.5, 0.1, 1e-2 – 1e-7,0 |
| Support vector machine | kernlab  (v. 0.9-31) [35] | *kernel* (kernel function)  *C* (cost of constraint violation)  *degree* (degree of polynomial kernel)  *scale* (scaling of polynomial kernel)  *sigma* (inverse width of radial kernel) | linear, polynomial, radial  0.001, 0.01, 0.1,1,10,100  2, 3, 4  0.1, 0.5, 1, 2  0.01, 0.1, 0.5, 1, 5, 10 |

Suppl. Table S2: Overview of machine learning (ML) algorithms. This table lists the ML algorithms and corresponding R packages that were employed to train MIBC vs. NMIBC classification models for the four miRNAs. Additionally, the model hyperparameters (as named in the respective package) including a brief description and the values that were tested in the 5-fold CV are provided.
