# Supplementary material for: Machine Learning Accurately Predicts Muscle Invasion of Bladder Cancer Based on Three miRNAs
Source: J Cell Mol Med. 2025 Feb 10;29(3):e70361. doi: 10.1111/jcmm.70361 (PMC11810526; doi:10.1111/jcmm.70361)
Supplement: Supplementary file 13 — Table S3. Results obtained by receiver operating characteristic (ROC) curve statistics; AUC, area under the curve. [file JCMM-29-e70361-s014.docx]

| **miR-138-5p** | **AUC** | **p-value** |
| --- | --- | --- |
| **Cohort 1** |  |  |
| TURB | 0.58 | 0.366 |
| CYS | 0.79 | **0.0005** |
| **Cohort 2** |  |  |
| TURB | 0.59 | 0.27 |
| CYS | 0.71 | **<0.0001** |

| **miR-146b-5p** | **AUC** | **p-value** |
| --- | --- | --- |
| **Cohort 1** |  |  |
| TURB | 0.90 | **<0.0001** |
| CYS | 0.98 | **<0.0001** |
| **Cohort 2** |  |  |
| TURB | 0.91 | **<0.0001** |
| CYS | 0.98 | **<0.0001** |

| **miR-155-5p** | **AUC** | **p-value** |
| --- | --- | --- |
| **Cohort 1** |  |  |
| TURB | 0.83 | **0.0004** |
| CYS | 0.87 | **<0.0001** |
| **Cohort 2** |  |  |
| TURB | 0.94 | **<0.0001** |
| CYS | 0.94 | **<0.0001** |

| **miR-200a-3p** | **AUC** | **p-value** |
| --- | --- | --- |
| **Cohort 1** |  |  |
| TURB | 0.64 | 0.119 |
| CYS | 0.78 | **0.0008** |
| **Cohort 2** |  |  |
| TURB | 0.56 | 0.426 |
| CYS | 0.73 | **<0.0001** |

Suppl. Table S3: Results obtained by receiver operating characteristic (ROC) curve statistics; AUC:_area under the curve.
