# Supplementary material for: Machine Learning Accurately Predicts Muscle Invasion of Bladder Cancer Based on Three miRNAs
Source: J Cell Mol Med. 2025 Feb 10;29(3):e70361. doi: 10.1111/jcmm.70361 (PMC11810526; doi:10.1111/jcmm.70361)
Supplement: Supplementary file 14 — Table S4. Prediction of muscle invasiveness in TURB pT1 hg tumour samples using the KNN model and conformal prediction (CP) and final histology in cystectomy (CYS) tumour samples. [file JCMM-29-e70361-s001.docx]

| **ID** | **TURB** | **CYS** | **KNN model** |
| --- | --- | --- | --- |
| 1 | pT1 hg | pT1/pTisG3pN0 | MIBC |
| 2 | pT1 hg | pT1G3 | MIBC |
| 3 | pT1 hg | pT1G3pN0 | MIBC |
| 4 | pT1 hg | pT1G3pN0 | MIBC |
| 5 | pT1 hg | pTispN0 | MIBC |
| 6 | pT1 hg | pT1G3pN0 mM | MIBC |
| 7 | pT1 hg | pT2bG3pN2 | MIBC |
| 8 | pT1 hg | pT2G3 pN0 | MIBC |
| 9 | pT1 hg | pT3aG3pN0 M1 | MIBC |
| 10 | pT1 hg | pT3aG3pN0L1 | MIBC |
| 11 | pT1 hg | pT3bG3pN0 | pTa lg |
| 12 | pT1 hg | pT4aG3pN2 | MIBC |

Suppl. Table S4: Prediction of muscle invasiveness in TURB pT1 hg tumor samples using the KNN model and conformal prediction (CP) and final histology in cystectomy (CYS) tumor samples
